# A Novel Study of Comorbidity between Schizoaffective Disorder and Geschwind Syndrome

**DOI:** 10.1155/2013/486064

**Published:** 2013-05-23

**Authors:** Kara O'Connell, Joanne Keaveney, Raymond Paul

**Affiliations:** ^1^Department of Psychiatry, Our Lady's Hospital, Navan, County Meath, Ireland; ^2^Department of Physiology, School of Medicine, Trinity College Institute of Neuroscience, Trinity College, Dublin, Ireland

## Abstract

Geschwind syndrome has been described in patients with temporal lobe epilepsy and is characterized by sexual behavioural disorders, hyperreligiosity, hypergraphia, and viscosity. Presented here is a case of a 53-year-old man with clinical findings consistent with Geschwind syndrome in the setting of a known diagnosis of schizoaffective disorder, with no identifiable comorbid illness of temporal lobe epilepsy or frontotemporal dementia. Brain MRI showed bilateral temporal lobe atrophy greater than would be expected for age and more prominent on the left side than the right. It is likely that these structural abnormalities may be related to this patient's clinical presentation of Geschwind syndrome. To our knowledge, this is the first reporting of a case of Geschwind syndrome in the setting of schizoaffective disorder. These symptoms of Geschwind syndrome were present irrespective of mental state status. The report highlights the importance in correct identification of underlying cause and differentiation between Geschwind syndrome and schizoaffective disorder in order to avoid mistreatment and consequent iatrogenic adverse events.

## 1. Introduction

Geschwind syndrome is an eponymous syndrome of interictal behaviour or personality disorder, which has been described in temporal lobe epilepsy [1, 2]. It has also been described in frontotemporal dementia [3]. Clinical features of this syndrome include preoccupation with philosophical and religious concerns, anger, excessive emotionality, viscosity (noted especially in speech), circumstantiality, altered sexuality, and hypergraphia. Recent reviews state that personality traits, rather than a personality disorder *per se*, seem more likely in these disorders, and they tend to resemble the cluster C category of disorders in DSM-IV [1, 2]. Schizoaffective disorder is episodic in which both affective and schizophrenic symptoms are prominent within the same episode of illness, preferably simultaneously but at least within a few days of each other. Patients who suffer from recurrent schizoaffective episodes, particularly those whose symptoms are of the manic rather than depressive type, usually make a full recovery and only rarely develop a defect state. Clinical presentations of schizoaffective disorder can include manic and depressive type states. It may therefore be difficult for clinicians to distinguish symptoms of Geschwind syndrome and those of schizoaffective manic type presentation, which can manifest as grandiose beliefs, religious delusions, and over activity.

We present a novel case of a 53-year-old man with clinical findings consistent with Geschwind syndrome in the setting of a known diagnosis of schizoaffective disorder.

## 2. Case

The patient is a 53-year-old single gentleman with a diagnosis of schizoaffective disorder, first identified at the age of 20 years. He had approximately twenty in-patient admission for both depressive and manic type episodes. The patient has been treated with various antipsychotic medications including clozapine, to which he experienced neutropenia, requiring cessation of treatment. He is presently maintained on olanzapine, lithium, and valproic acid medications. The patient currently presents with long-standing hypergraphia, religious preoccupation, and hyposexuality. An example of these symptoms is the patient's preoccupation with God, writing multiple pages daily stating, “God is good, God is good.” These symptoms have been present irrespective of mental state status. The patient stated that he has never had nor is interested in a sexual relationship and describes an absent libido, suggestive of hyposexuality. He is not experiencing delusions, affective disturbance, hallucinations or any biological symptoms of a mental disorder at present. This patient does not have a diagnosis of frontotemporal dementia or epilepsy. 

In psychometric testing, the patient's overall IQ scored 103, with deficits being displayed in semantic fluency, though categorization was intact. The patient obtained very low scores on two verbal memory subtests of the Rivermead Behavioural Memory Test-Third Edition (RBMT-3): Story-Immediate Recall and Story-Delayed Recall. Lesions in the left temporal lobe are associated with poor verbal memory scores. He obtained a raw score of 11 on the Repeatable Battery for the Assessment of Neuropsychological Status (RBANS) Semantic Fluency subtest (the mean score obtained by the standardization sample aged 50–59 on this subtest was 21). There was no evidence of frontotemporal dementia. No premorbid testing had been undertaken.

The patient had no past significant medical history. He had no known history of developmental delay, obstetric complications, or injury at birth. Neither the patient nor his family described any current or past clinical, neurological, or other symptoms suggestive of epilepsy. He also has no known history of illicit drug or alcohol use. Physical examination was unremarkable with no focal neurological signs evident. An EEG identified intermittent disturbance of right and left temporal function, but these were nonspecific and not characteristic of epileptic form activity. An MRI brain scan showed bilateral temporal lobe atrophy greater than would be expected at his age and more prominent on the left side than the right. The hippocampus was normal. There was noted bilateral widening of choroidal fissures. Blood investigations (including full blood count, urea and electrolytes, and liver function tests) were normal.

## 3. Discussion

A search of Pubmed using the search terms “Geschwind” in combination with “schizoaffective disorder” or “bipolar affective disorder” displayed no matching articles. This is the first time that a case of Geschwind syndrome has been reported in the setting of schizoaffective disorder and in the absence of a known comorbid diagnosis of temporal lobe epilepsy or frontotemporal dementia.

A study of temporal lobe epilepsy patients has shown that while bilateral symmetrical hippocampal atrophy does not result in an increased prevalence of specific psychiatric syndromes, specific symptoms characteristic of the Geschwind syndrome, like hypergraphia and hyposexuality, might be pathogenically related to hippocampal atrophy [4]. Notably, there was no hippocampal atrophy identified on MRI brain in this case. In the case we are presenting, neuropsychological testing provided no evidence of frontotemporal dementia. The patient had no identifiable personality disorder. He exhibited poor semantic fluency, which is known to be associated with temporal lobe damage; he was able, however, to demonstrate semantic categorization.

Neuropsychiatric symptoms are common manifestations of temporolimbic lesions. Temporolimbic networks interface with multiple cortical and subcortical circuits that modulate emotional behavior and affect potentially giving rise to the Geschwind syndrome [5]. A case series has been presented that identified organic temporal lobe abnormalities in all cases of hypergraphia studied [6]. Hypergraphia reflects changes in emotional responsiveness secondary to organic temporal lobe lesions. It has previously been suggested that there is a close relationship between emotional difficulties and hypergraphia [6]. According to Geschwind the interictal symptoms are associated with an intermittent spike focus in the temporal lobe, leading to altered responsiveness of the limbic system [7]. This alteration is manifested as deepened emotionality and hypergraphia.

## 4. Conclusion

It is important to note the possibility of occurrence of symptoms such as hyperreligiosity, emotionality, and hypergraphia and to acknowledge that they may not be illness state specific. Therefore, clinicians should be mindful regarding diagnosis and treatment of patients presenting with symptoms suggestive of schizoaffective disorders. This may avoid unnecessary pharmacological treatment, which could result in potential increased risks of adverse events and side effects, without clinical gain. In this gentleman's case, if Geschwind syndrome had not been identified, he may be subjected to even higher doses of medication with undesirable side effects and no benefit being derived.
